# Two-Photon Laser Ablation and In Vivo Wide-Field Imaging of Inferior Olive Neurons Revealed the Recovery of Olivocerebellar Circuits in Zebrafish

**DOI:** 10.3390/ijerph18168357

**Published:** 2021-08-06

**Authors:** Kanae Hiyoshi, Kaito Saito, Narumi Fukuda, Takahisa Matsuzaki, Hiroshi Y. Yoshikawa, Sachiko Tsuda

**Affiliations:** 1 Division of Life Science, Graduate School of Science and Engineering, Saitama University, Saitama 338-8570, Japan; k.hiyoshi.785@ms.saitama-u.ac.jp (K.H.); k.saito.644@ms.saitama-u.ac.jp (K.S.); kakeru3352@gmail.com (N.F.); 2 Division of Strategic Research and Development, Graduate School of Science and Engineering, Saitama University, Saitama 338-8570, Japan; taka.chem2@gmail.com (T.M.); hiroshi@ap.eng.osaka-u.ac.jp (H.Y.Y.); 3 Department of Chemistry, Graduate School of Science and Engineering, Saitama University, Saitama 338-8570, Japan; 4 Department of Applied Physics, Graduate School of Engineering, Osaka University, Suita 565-0871, Japan; 5 Integrative Research Center for Life Sciences and Biotechnology, Saitama University, Saitama 338-8570, Japan

**Keywords:** two-photon laser ablation, in vivo imaging, zebrafish, cerebellar reserve, olivocerebellar circuit, inferior olive, climbing fiber

## Abstract

The cerebellum, a brain region with a high degree of plasticity, is pivotal in motor control, learning, and cognition. The cerebellar reserve is the capacity of the cerebellum to respond and adapt to various disorders via resilience and reversibility. Although structural and functional recovery has been reported in mammals and has attracted attention regarding treatments for cerebellar dysfunction, such as spinocerebellar degeneration, the regulatory mechanisms of the cerebellar reserve are largely unidentified, particularly at the circuit level. Herein, we established an optical approach using zebrafish, an ideal vertebrate model in optical techniques, neuroscience, and developmental biology. By combining two-photon laser ablation of the inferior olive (IO) and long-term non-invasive imaging of “the whole brain” at a single-cell resolution, we succeeded in visualization of the morphological changes occurring in the IO neuron population and showed at a single-cell level that structural remodeling of the olivocerebellar circuit occurred in a relatively short period. This system, in combination with various functional analyses, represents a novel and powerful approach for uncovering the mechanisms of the cerebellar reserve, and highlights the potential of the zebrafish model to elucidate the organizing principles of neuronal circuits and their homeostasis in health and disease.

## 1. Introduction

A remarkable aspect of the brain is its plasticity, which is essential for brain development, function, and homeostasis [1,2,3,4,5,6]. As a brain region with a high degree of plasticity, the cerebellum plays pivotal roles in motor control and learning, as well as in cognition [7,8,9,10]. A collection of anatomical and physiological studies have led to numerous insights regarding information processing in the cerebellum, especially regarding synaptic plasticity and learning [11,12,13,14].

A prominent example of cerebellar plasticity is the “cerebellar reserve”, which is the capacity of the cerebellum to compensate and restore function to adapt to injury or other disorders via resistance and plasticity [15]. For example, motor dysfunction caused by a cerebellectomy or injury could gradually be compensated for and restored [15,16,17,18,19,20]. This is particularly interesting in the context of spinocerebellar degeneration (SCD), which is a progressive condition for which an effective treatment has not been established [21,22]. Regarding the process of recovery, it has been proposed that cerebellar function could be restored with appropriate treatment only in the restorable stage, when the cerebellar function is still well preserved [23,24]. Therefore, information on the regulatory mechanisms of the cerebellar reserve is critical. However, these mechanisms are largely unidentified, particularly at the circuit level. Moreover, previous studies have suggested that the cerebellar reserve is plastic and modulated by environmental factors, such as enriched environments (EEs) [15,25,26,27]. The variables affecting this phenomenon have yet to be identified.

**Figure 1:**
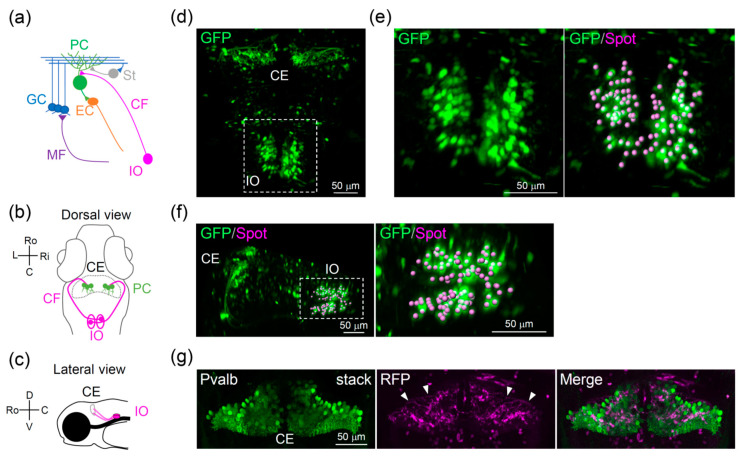
Cerebellar circuits in zebrafish: (**a**) Schematic diagram of the cerebellar circuits in zebrafish. (**b**,**c**) Schematic diagram of the olivocerebellar circuits: (**b**) Dorsal view and (**c**) lateral view. (**d**–**f**) Distribution of inferior olive neurons in *Tg*(*hspGFFDMC28C*;*UAS*:*GFP)* zebrafish at 7 days post-fertilization (dpf): (**d**,**e**) Dorsal view, with a high-magnification image shown in (**e**), where spots indicate the position of the soma of inferior olive neurons. (**f**) Dorsolateral view, with a high-magnification image of the inferior olive shown in the right panel. (**g**) Dorsal view of the cerebellum of *Tg*(*hspGFFDMC28C*;*UAS*:*RFP)* larva stained with Parvalbumin 7 (Pvalb) at 6 dpf (confocal z-stack images). Arrowheads indicate CFs. CE, cerebellum; CF, climbing fiber; EC, eurydendroid cell; GC, granule cell; IO, inferior olive; MF, mossy fiber; PC, Purkinje cell; St, stellate cell; Ro, rostral; C, cordal; L, left; Ri, right; D, dorsal; V, ventral.

In order to solve these issues, the optical control and detection of neurons provide a powerful approach facilitating the examination of neuronal population dynamics [28]. By using light, neurons of interest can easily be accessed non-invasively in a wide field, which is critical to analyze network dynamics in the brain. In the case of the cerebellar reserve, it is important to characterize the structural and functional changes that occur in a wide area of the brain in the context of recovery. However, such a strategy is unavailable; this is because long-term, non-invasive observation of the whole cerebellar circuits is difficult to be performed in mammalian systems due, in part, to the large size and opacity of the mammalian brain [29]. Herein, we used zebrafish to develop a novel system to enable the optical detection of changes in the cerebellar circuits in response to optical lesions. Zebrafish are small vertebrates used in many research fields, including developmental biology, neuroscience, and medical science, with particular advantages for optical approaches and genetic methods [30,31,32]. The transparency and small size of zebrafish make them suitable for non-invasive live imaging, particularly for the whole-brain application of optical techniques in circuit analysis [33,34,35]. As for the cerebellum, zebrafish are known to have cerebellar circuits that are structurally and functionally similar to mammalian ones, and most of the major neuron types in the cerebellum have already been identified [36,37] (Figure 1a). Furthermore, transgenic zebrafish lines that express *gal4* in a neuron-type-specific manner are available for most of the major neuron types in the cerebellum, providing an excellent system for circuit analysis [38]. Recent studies using whole-cerebellar functional imaging, such as calcium and voltage imaging, have uncovered the functional organization of zebrafish cerebellar circuits involved in various modalities, including sensorimotor integration and emotion [39,40,41,42,43]. Cerebellar dysfunction, such as ataxia and tremor, can be evaluated by behavioral properties, including swimming patterns and eye movement [44,45]. Adaptive properties of the zebrafish cerebellum have also been identified, such as those implicated in motor adaptation and fear learning [33,43,46]. However, the cerebellar reserve has not been examined in zebrafish.

Herein, we sought to develop a novel optical system in zebrafish that enables long-term and non-invasive circuit analysis to uncover the mechanisms of the cerebellar reserve at the neuronal circuit level. For this purpose, we combined two-photon laser ablation of the inferior olive (IO) and in vivo whole-brain imaging. We focused on the IO because this is one of the regions critical for cerebellar function and adaptation [47,48]. The IO sends key inputs, such as error signals in motor learning, to the cerebellum through olivocerebellar circuits, which are a major site for circuit rearrangement during development [47,49]. Furthermore, the IO is located in the brainstem, which is relatively distant from the cerebellum (Figure 1b,c). Thus, it is possible to manipulate the IO without causing any direct damage to the cerebellum. Laser irradiation of IO neurons in transgenic zebrafish, in which IO neurons were labeled by red fluorescent protein (RFP), led to severe damage in IO neurons. This included the loss of climbing fibers (CFs), the axons of IO neurons. In vivo, wide-field imaging of the ablated fish showed that olivocerebellar circuit recovery began shortly after IO ablation, i.e., in less than a week. These results indicate that we succeeded in optically inducing acute lesions in the zebrafish IO, and that the recovery process of the olivocerebellar circuit could be visualized in vivo in a non-invasive manner. Thus, this system provides a novel and powerful approach for uncovering the mechanisms of the cerebellar reserve, and highlights the potential of the zebrafish model for elucidating the organizing principles of neuronal circuits and their homeostasis.

## 2. Materials and Methods

### 2.1. Fish and Transgenic Lines

We used transgenic zebrafish lines *Tg*(*hspGFFDMC28C*;*UAS*:*RFP)* and *Tg*(*hspGFFDMC28C*;*UAS*:*GFP)* that express RFP and GFP in IO neurons, respectively [38]. Adult zebrafish were maintained in a room with a 14 h light/10 h dark cycle. For most of the experiments, the embryos were incubated at 28 °C. All experiments using live fish complied with the protocols approved by the Committee for Animal Care and Use of Saitama University (R3-A-1-5).

### 2.2. Two-Photon Laser Ablation

Zebrafish larvae (*Tg*(*hspGFFDMC28C*;*UAS*:*RFP)*, six days post-fertilization (dpf)) were immobilized in 2% low-melting point agarose (Sigma-Aldrich, St. Louis, MO, USA). We used a femtosecond laser (λ = 780 nm, Δt ~70 fs, Chameleon, SpectraPhysics) to irradiate the soma of IO nuclei neurons on an inverted microscope (A1R MP+, Nikon, Plan Apoλ 40 × /NA 0.95). The target area was determined on the basis of the fluorescence signals (RFP) of the IO neurons, and the regions of interest (ROIs) were defined using an NIS-elements program (Nikon, Tokyo, Japan). Laser irradiation was repeated until the cells and/or tissues showed morphological defects and the RFP signals of the targeted IO neurons were absent. Only larvae that showed clear morphological defects in IO neurons were used for the following analysis. By irradiating two to three different focal planes along the dorsoventral axis of the zebrafish, the entire region containing the inferior olive was ablated. After laser ablation, the fish were incubated at 28 °C, and then we conducted confocal microscopy imaging.

### 2.3. Confocal Microscopy Imaging

Zebrafish larvae were paralyzed via 0.02% tricaine (Sigma-Aldrich), and then mounted on 2% low-melting point agarose. The specimens were observed using a confocal microscope (A1R, Nikon, Apo LWD 25 × /NA 1.1, FV1000, Olympus, UPL SAPO 40 × /NA 0.95).

### 2.4. Cell Counting

We measured the number of IO neurons labeled by green florescent protein (GFP) or RFP using an automatic technique (Imaris, Bitplane, Spot function). The soma of the IO neurons was detected using confocal XYZ scanned images. False-positives and missed cells were removed and added manually, respectively.

### 2.5. Whole-Mount Immunostaining

Zebrafish larvae were fixed at 4 °C overnight in 4% PFA in PBST (PBS, 0.1% Triton X-100). The samples were then washed with PBST and incubated in acetone at −20 °C. Larvae were washed once with PBST and PBSDT (PBS, 1% BSA, 1% DMSO, 1% Triton X-100), and incubated in 5% goat serum (Vector) in PBS-DT at room temperature (RT) for 2 h. The samples were incubated with the primary antibody solution (anti-zebrafish Parvalbumin7 antibody, 1/1000, a marker for Purkinje cells in zebrafish [38]) at 4 °C overnight. After six washes with PBST, the samples were incubated with the secondary antibody (Alexa Fluor 647 goat anti-mouse IgG antibody (Molecular Probes, 1/500)). Nuclei were counterstained with DAPI (Molecular Probes). These specimens were observed using a confocal microscope.

## 3. Results

### 3.1. Distribution of Inferior Olive Neurons in Zebrafish

To address the mechanisms of the cerebellar reserve with a focus on the olivocerebellar circuit (Figure 1a–c), we first examined the distribution of IO neurons in zebrafish. To this end, we used a transgenic fish line that expresses RFP in IO neurons (*Tg(hspGFFDMC28C;UAS:RFP*) [38]) at 6 dpf, which is when the basic structure of the cerebellar circuit has formed [36]. Confocal microscopy enabled the in vivo observation of the entire region containing the olivocerebellar circuit at a single-cell resolution, which indicated that IO neuron somas were distributed in clusters in the posterior hindbrain (Figure 1d–f). As previously reported, this zebrafish Tg line also expresses RFP in some non-IO neurons [38], but these could be easily distinguished and excluded from the following analysis. We next quantified the number of IO neurons via 3D spot analysis using 3D reconstructed images (see the Methods section). At 6–7 dpf, each IO contained approximately 80 neurons (77.0 ± 5.1 cells; 8 fish), which were distributed within an area with a width of approximately 100 μm. Immunohistochemical analysis showed that RFP-positive CFs were widely observed in the cerebellum in close proximity to parvalbumin-positive Purkinje cells (Figure 1g). The number of Purkinje cells (parvalbumin 7-positive) was also small, i.e., approximately 450 cells (457.2 ± 13.0 cells; 5 fish; 5 dpf). These results demonstrate that zebrafish have a much smaller number of IO neurons and Purkinje cells than mammals, which can be advantageous in this research [50].

### 3.2. Two-Photon Laser Ablation of IO Neurons

**Figure 2:**
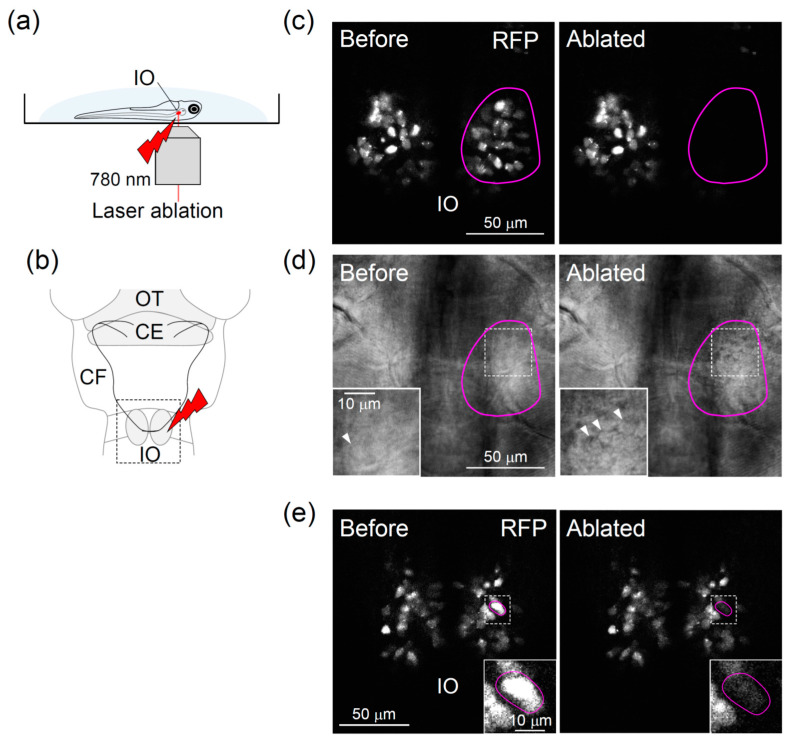
Two-photon laser ablation of the inferior olive in zebrafish: (**a**) Schematic diagram of the laser ablation system. (**b**) Right hemisphere of the inferior olive was irradiated. CE, cerebellum; CF, climbing fiber; IO, inferior olive; OT, optic tectum. (**c**–**e**) Images of the inferior olive neurons before (left) and shortly after (right) laser irradiation. Red circles indicate the target area of irradiation. The arrowheads in the magnified images in (**d**) indicate neurons that showed abnormal morphology after laser irradiation (**d**, right).

Targeting cells using light could enable the manipulation of cells at a precise location and time. Herein, we combined two-photon laser ablation of IO neurons with confocal long-term in vivo imaging. Because the IO is part of the brainstem, which is located deep in the brain, we used an infrared laser that produced light that could penetrate deep into the tissue compared with a single photon laser [51] (Figure 2a,b).

For the laser ablation of IO neurons, the IO neurons in the right hemisphere of the *Tg*(*hspGFFDMC28C*;*UAS*:*RFP)* fish were illuminated by an infrared laser. Confocal imaging showed that after laser irradiation, the RFP signals in the target area had almost completely disappeared (Figure 2c, 13 fish). Many IO neurons at the illuminated region showed abnormal morphology, such as swelling, indicating that the laser irradiation induced severe damage (Figure 2d, 13 fish). However, these defects were not observed in the opposite side of the IO, which had not been irradiated (Figure 2c,d). Furthermore, ablating a smaller number of neurons was also possible, leading to a lesion of the IO neurons at a single-cell resolution (Figure 2e). These results indicate that we succeeded in the laser ablation of IO neurons in zebrafish in vivo.

### 3.3. Recovery of the Olivocerebellar Circuit after IO Ablation

**Figure 3:**
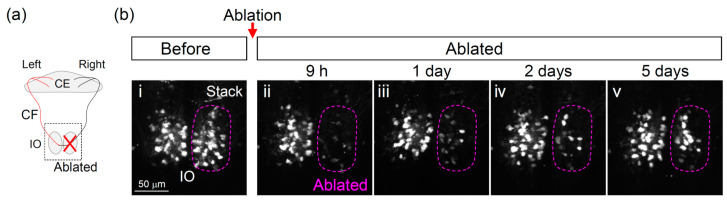
Changes in the inferior olive neurons after two-photon laser ablation: (**a**) Schematic diagram of the zebrafish inferior olive and cerebellum. CE: cerebellum, CF: climbing fiber, IO: inferior olive; (**b**) Dorsal view of the inferior olive (confocal z-stack images). The red dashed lines indicate the ablated region (the right hemisphere of the inferior olive). Before ablation: ⅰ, after ablation: ⅱ (9 h), iii (1 day), iv (2 days), and v (5 days).

**Figure 4:**
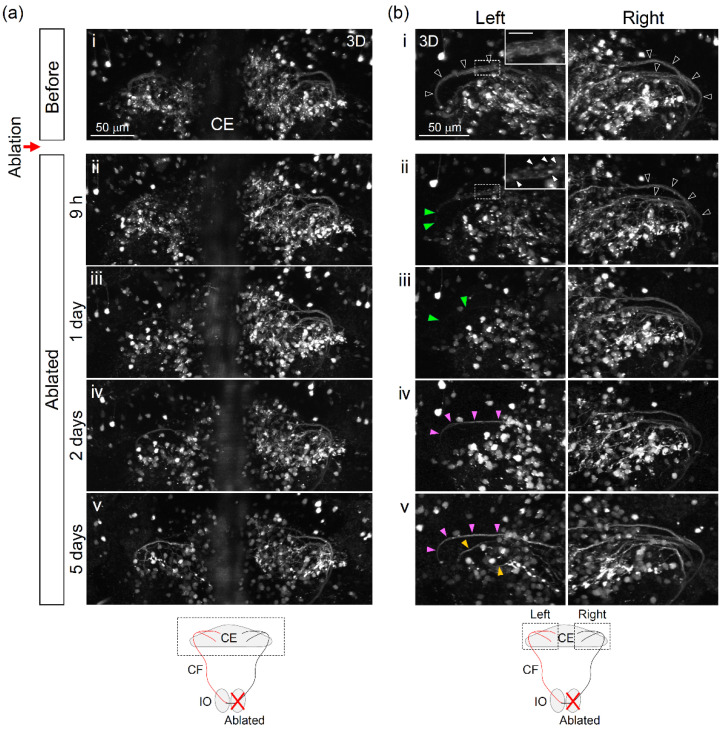
Structural changes of climbing fibers (CFs) in the cerebellar region induced by the two-photon laser ablation of the inferior olive. (**a**,**b**) Three-dimensional dorsal view of climbing fibers. The area of observations is shown by the dotted line in the schematic diagram. Before ablation: i, after ablation: ii (9 h), iii (1 day), iv (2 days), and v (5 days). (**b**) A similar region as (a), but the orientation is slightly different: A high-magnification image is inserted in the upper right corner in (**b-i**) (before) and (**b-ii**) (after 9 h). Scale bar: 10 μm. Black arrowheads in (**b-i**) indicate the bundle of CFs. Green arrowheads in (**b-ii,iii**) indicate the defected CFs after laser ablation. Red and yellow arrowheads (**b-iv,v**) indicate newly emerged CFs.

An advantage of the zebrafish model is that it allows long-term in vivo non-invasive observation of various organs throughout the body at a single-cell resolution. We next investigated the potential changes in the olivocerebellar circuit after IO ablation. We conducted two-photon laser ablation of a large population of IO neurons (right hemisphere, Figure 2c) in *Tg*(*hspGFFDMC28C*;*UAS*:*RFP)* fish at 6 dpf, and then observed the fish by confocal microscopy (at 0, 1, 2, and 4–5 days after laser ablation). In the following paragraphs, we describe the results obtained at the soma and axons (CFs) of the IO (Figure 3 and Figure 4).

In the ablated side of the IO, the number of somas of IO neurons (RFP-positive) markedly decreased at 9 h after ablation (6/6 fish, Figure 3bii). In contrast, the IO on the contralateral side showed no change. Subsequent analysis indicated that the number of IO neurons in the ablated side gradually increased (Figure 3bii–v), suggesting partial recovery of the neurons in this area. Indeed, four to five days after the ablation, most fish exhibited an increased number of RFP-positive IO cells compared to immediately after ablation (4/6 fish, Figure 3bv). In the IO on the opposite side of the ablation, we saw no clear change.

Because IO neurons project to the contralateral side of the cerebellum (Figure 4a) [38], we expected that the CFs in the left hemisphere would exhibit abnormalities. Confocal imaging of the olivocerebellar circuit showed that, approximately 9 h after the ablation, CFs in the left hemisphere were disrupted, and some of the axon bundles disappeared (5/6 fish, Figure 4aii,iii,bii,iii, green arrowheads). In addition, aggregation was observed in the cerebellum (Figure 4aii,bii, white arrowheads). This aggregation gradually disappeared over two days. Interestingly, two days after the ablation, a long bundle of axons appeared in the cerebellum (Figure 4biv,v, red arrowheads). These might be CFs that were recovered after the lesion. Furthermore, at four to five days after the ablation, we observed thin fibers that extended from the thick bundle of axons outward in various directions (Figure 4bv, yellow arrowhead). In the right hemisphere of the IO, however, such clear changes were not observed. These results suggest that although the CFs were severely damaged after the IO ablation, they could partially recover in less than a week.

## 4. Discussion

In the present study, we developed a novel optical approach that enables (1) optical lesions in the inferior olive and (2) visualization of circuit remodeling non-invasively in a wide field of the brain using the zebrafish model. This type of analysis is impossible to perform in the mammalian system; thus, our system provides a powerful and novel approach for cerebellar research, especially for analyzing the cerebellar reserve.

In vivo analysis of the brain circuits in mammals is often hampered by the large size and opacity of the brain, as well as the numerous neurons. This is also the case in the cerebellum; for example, the mammalian IO comprises approximately 8600 neurons, which are difficult to thoroughly examine in vivo [50]. In contrast, in zebrafish, we showed that the IO contains approximately 80 neurons only, enabling in vivo live imaging of the entire neural population in the IO.

In this study, we utilized two-photon (infrared) laser ablation to induce acute lesions in zebrafish IO, which was followed by long-term in vivo imaging. Compared to other approaches, such as cerebellectomy [52,53] and drug application (cf. kainic acid, 3-acetylpyridine [54,55]), this optical lesion allows for the ablation of neurons at desirable locations and timing at a high spatial resolution (single-cell level). Moreover, because infrared light can penetrate deep within the tissue without damaging the cells located away from the focal point, two-photon laser ablation is suitable for inducing acute lesions in vivo (Table S1). By combining the powerful genetics of zebrafish, we could perform ablation in a cell-type specific manner, providing an optimal system for analyzing the cerebellar reserve. Regarding the recovery process of the neuronal circuits after an IO lesion, in mammals, it is challenging to analyze the changes in neuronal circuits at a single-cell level in vivo, partly because of the invasiveness of the observation. In contrast, in our novel system with zebrafish, it was easy to observe the post-lesion process non-invasively, and for a long time at the level of a single cell. Moreover, the morphological analysis reported herein can easily be combined with functional analysis of the neuronal circuits in zebrafish, such as calcium/voltage imaging, optogenetics, and behavioral assays [35,41,42,56,57]. Therefore, zebrafish provide a unique system for uncovering the structural and functional plasticity that underlies the cerebellar reserve at the network level at high resolution.

In our study, laser irradiation of a hemisphere of the IO caused severe morphological deficits in IO neurons in a relatively short period of time (several hours to one day), which was observed both in the soma and the axons (CFs) of the irradiated IO neurons. In CFs, aggregation was observed widely in the cerebellum. These aggregations may be indicative of the degradation process of the damaged CFs. This rather rapid degeneration of the inferior olive neurons is similar to the previous finding in rat IO induced by the administration of 3-acetylpyridine [54]. Within less than a week of IO ablation, the olivocerebellar circuits showed significant structural remodeling and partial recovery. This rather fast process of recovery might be explained by the rapid extension of axons in zebrafish. Considering the rate of zebrafish axon elongation of about 15–20 μm/h [58], we could estimate that it might take less than a day for IO neurons to project to the cerebellum, which is approximately 300 μm away from the IO. There is a possibility that the rate of recovery might differ among IO neurons dependent on the degree of damage. A more detailed analysis of these neurons would help understand the contribution of each cell. Future studies using time-lapse imaging of CF extension and ablation of less IO neurons would provide more details about this process. Furthermore, it has been shown that the process of recovery by the cerebellar reserve differs depending on the timing of the lesions. The rat cerebellum lesioned in the neonatal period exhibited more prominent recovery in approximately three months than one lesioned in adulthood [18,19,20,59]. As we used zebrafish larvae in our current study, the remodeling process might also be facilitated, which would even enhance the advantages of our experimental system.

With the visualization of the structural recovery process of the IO neurons achieved in this study, the next important step is to analyze the potential functional changes of the IO neurons, as well as those of the other neurons constituting olivocerebellar circuits, and also glia [6,54]. Further investigation, together with a variety of functional analyses of neural networks available in zebrafish (as mentioned above), would help understanding the mechanism of the cerebellar reserve at the neural circuit level. Indeed, previous studies have shown that IO lesions cause functional abnormalities, including cerebellum-dependent motor learning in mammals and zebrafish [33,60,61]. Thus, it is plausible that the structural remodeling observed herein might be parallel to functional remodeling.

The optical system established in this study could provide powerful disease models for various cerebellar disorders, including spinocerebellar degeneration and essential tremor [45,62]. There is a debate regarding the functional role of the IO in essential tremors [63]. Our experimental system would greatly help arrive at a conclusion on this issue. Various neuron types have been reported to show degeneration, leading to severe ataxia and, in some cases, mental disorders [64,65]. Our optical approach can also be applied to these other neuron types in the cerebellum to elucidate the pathogenesis of SCD at the cellular and neuronal circuit levels. Furthermore, although we used a large-scale lesion in our current study, the position and number of target cells for ablation can easily be controlled by utilizing light. This would allow for a quantitative approach to examine the key properties of the cerebellar reserve and plasticity, for example, whether the cerebellum could be recovered after a certain amount of damage. Such information would be valuable for understanding the mechanisms of the cerebellar reserve, such as the level of damage that allows recovery of cerebellar function, where the boundary between restorable and non-restorable stages lie, and how this boundary is determined and controlled. Functional compartmentalization of the IO and olivocerebellar circuits would also need to be considered [66,67,68].

There are, however, some limitations. Although the structure and function of the cerebellum and its developmental process have been identified in zebrafish, further examination of its anatomy and functional organization of the cerebellar circuits are necessary. For instance, detailed anatomical information of the IO and innervations to the IO remained to be elucidated [69]. The genetic resources of zebrafish and advantages in optics, along with the use of the cumulated information in mammals, would help solve this issue [30,31,32,38].

In zebrafish, functional development, including for sensory, motor, and social systems, is known to mature quickly—within less than a week. With the already established experimental system for environmental enrichment research in zebrafish [70,71,72,73], it would be of great interest to examine the influence of external conditions on the cerebellar reserve in the context of neuronal circuits.

## 5. Conclusions

We developed an optical approach that enables in vivo characterization of structural remodeling of cerebellar networks using zebrafish. By combining two-photon laser ablation and long-term in vivo wide-field imaging of the entire population of IO neurons, we succeeded in visualizing changes in the olivocerebellar circuit in response to an inferior olive lesion. This system, in combination with various functional analyses, provides a novel and powerful approach for uncovering the mechanisms of the cerebellar reserve, and highlights the potential of the zebrafish model to elucidate the organizing principles of neuronal circuits and their homeostasis in health and disease.

## Data Availability

The data presented in this study are available on request from the corresponding author.

## Supplementary Materials

The following are available online at https://www.mdpi.com/article/10.3390/ijerph18168357/s1. Table S1: Approaches for cerebellar lesions and their advantages.
