# Supplementary material for: Two-Photon Laser Ablation and In Vivo Wide-Field Imaging of Inferior Olive Neurons Revealed the Recovery of Olivocerebellar Circuits in Zebrafish
Source: Int J Environ Res Public Health. 2021 Aug 6;18(16):8357. doi: 10.3390/ijerph18168357 (PMC8391264; doi:10.3390/ijerph18168357)
Supplement: Supplementary file 1 [file ijerph-18-08357-s001.zip › ijerph-1292392-supplementary.pdf]

Approach	Advantages
Cerebellectomy	Relatively simple procedure, cells are physically removed
Drug application	Chemical targeting is possible, easily applicable to deep brain
Laser ablation	High spatiotemporal resolution, less invasive, easily applicable to deep brain

Table S1 Approaches for cerebellar lesion and their advantages
